# Supplementary figures and images for: Aminomethanesulfonic acid illuminates the boundary between full and partial agonists of the pentameric glycine receptor
Source: eLife. 2022 Aug 17;11:e79148. doi: 10.7554/eLife.79148 (PMC9462852; doi:10.7554/eLife.79148)

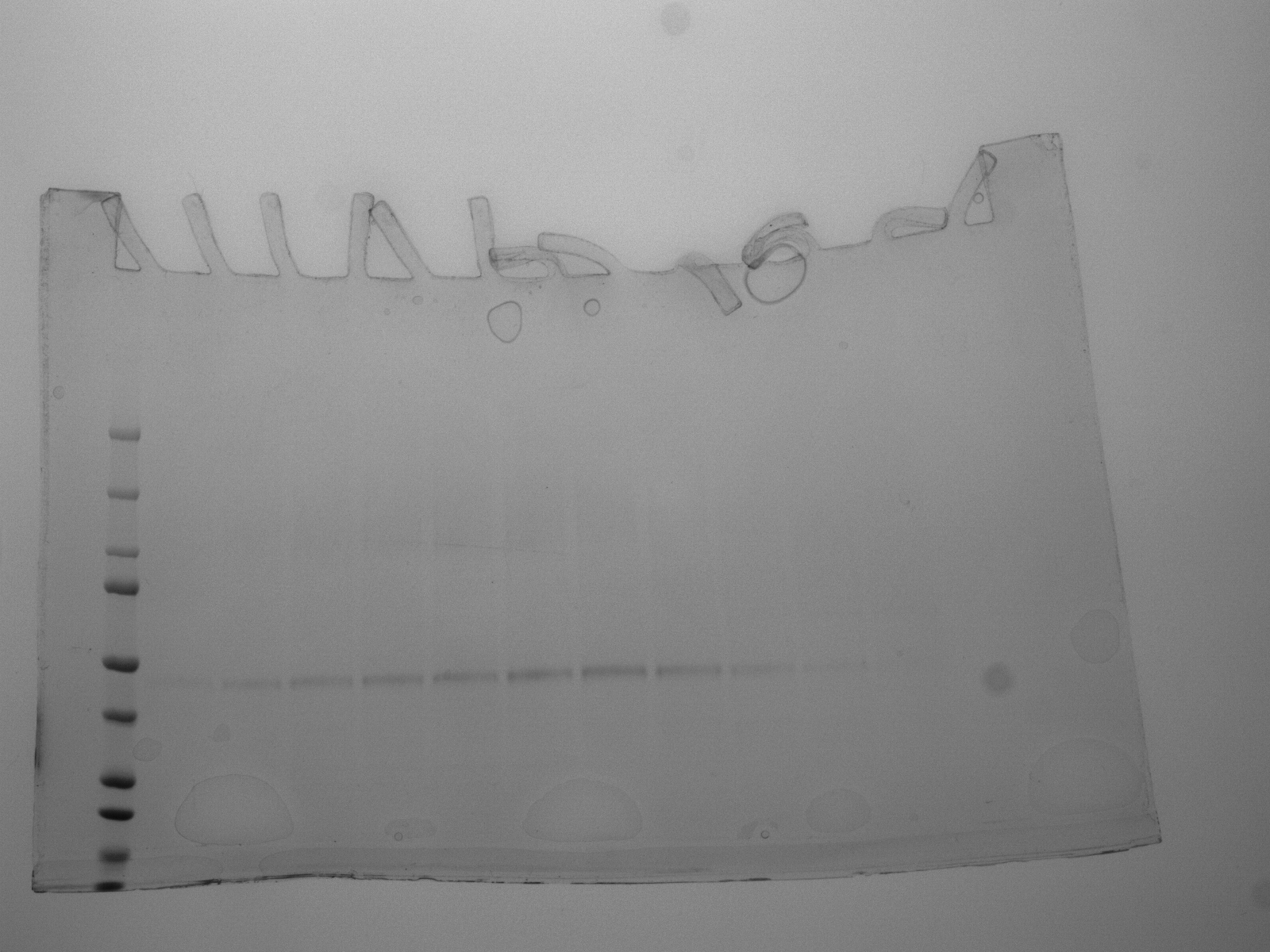

Supplement: Figure 3—figure supplement 1—source data 1. [file elife-79148-fig3-figsupp1-data1.zip › Source data/Figure3-figrue supplement 1-source data 1.TIF]

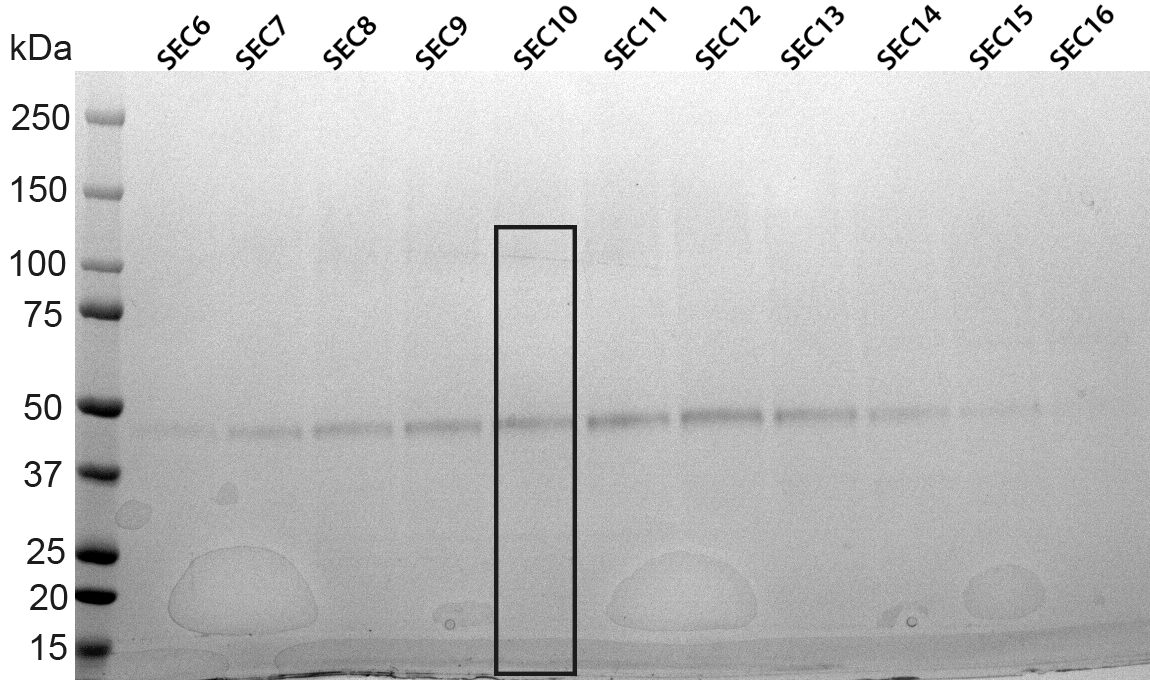

Supplement: Figure 3—figure supplement 1—source data 1. [file elife-79148-fig3-figsupp1-data1.zip › Source data/Figure3-figrue supplement 1-source data 2.tif]
