# Supplementary material for: Aminomethanesulfonic acid illuminates the boundary between full and partial agonists of the pentameric glycine receptor
Source: eLife. 2022 Aug 17;11:e79148. doi: 10.7554/eLife.79148 (PMC9462852; doi:10.7554/eLife.79148)
Supplement: Supplementary file 1. [file elife-79148-supp1.docx]

**Extended Data Table 1 Statistics for 3D reconstruction and model refinement.**

| **States** | **Open** | **Desensitized** | **Expanded-Open** |
| --- | --- | --- | --- |
| **Codes** | (EMD-26316)  (PDB-7U2N) | (EMD-26315)  (PDB-7U2M) | (EMD-26317)  (PDB-7U2O) |
| **Data collection and processing** | | | |
| Microscope | Titan Krios | | |
| Camera | K3 BioQuantum | | |
| Magnification | 105,000 | | |
| Voltage (kV) | 300 | | |
| Defocus range (μm) | -1.2 to -2.2 | | |
| Exposure time (s) | 4.3 | | |
| Dose rate (*e^-^*/Å^2^/s) | 8 | | |
| Number of frames | 50 | | |
| Pixel size (Å) | 0.831 | | |
| Micrographs (no.) | 12,179 | | |
| Initial particles (no.) | 936,714 | | |
| Symmetry imposed | C5 | | |
| Final particles (no.) | 207,486 | 7,414 | 70,616 |
| Map resolution (Å) | 2.8 | 2.9 | 3.1 |
| FSC threshold | 0.143 | 0.143 | 0.143 |
| **Refinement** | | | |
| Initial model (PDB code) | 6PM2 | 6PM1 | 6PM0 |
| Model resolution (Å) | 2.9 | 3.0 | 3.2 |
| FSC threshold | 0.5 | 0.5 | 0.5 |
| Model composition | | | |
| Non-hydrogen atoms | 14100 | 14,175 | 14095 |
| Protein atoms | 13,855 | 13,930 | 13,850 |
| Ligand atoms | 245 | 245 | 245 |
| *B* factors (Å^2^) | | | |
| Protein | 97.2 | 102.9 | 97.7 |
| Ligand | 76.7 | 76.7 | 75.4 |
| R.m.s. deviations | | | |
| Bond length (Å) | 0.007 | 0.008 | 0.006 |
| Bond angle (˚) | 0.695 | 0.744 | 0.691 |
| **Validation** | | | |
| Favored (%) | 91.44 | 94.56 | 93.86 |
| Allowed (%) | 8.56 | 5.44 | 6.14 |
| Disallowed (%) | 0 | 0 | 0 |
| Poor rotamers | 0 | 0 | 0 |
| MolProbity score | 1.94 | 1.72 | 1.93 |
| Clash score | 7.94 | 6.35 | 10.07 |
